# A Review of the Use of Biological Agents in Human Immunodeficiency Virus Positive Patients With Rheumatological Diseases

**DOI:** 10.7759/cureus.10970

**Published:** 2020-10-15

**Authors:** Marline A Attallah, Maria Daniela Jarrin Jara, Avneesh S Gautam, Venkata Sri Ramani Peesapati, Safeera Khan

**Affiliations:** 1 Internal Medicine, California Institute of Behavioral Neurosciences and Psychology, Fairfield, USA; 2 Medicine and Surgery, Bharati Vidyapeeth Medical College, Pune, IND

**Keywords:** hiv, rheumatological, biological agents

## Abstract

After approval, initial biologics etanercept, infliximab, and adalimumab became useful in the therapeutic armamentarium to treat rheumatoid arthritis (RA) patients who had an inadequate response to disease-modifying anti-rheumatic drugs (DMARDs). However, all phase-III clinical trials submitted to the FDA, by design, excluded patients who were human immunodeficiency virus (HIV) positive. They are another subset of patients with low immunity due to their HIV-positive status. Very little information is available about the use of biologics in this new group of patients if they fail to respond to DMARDS. The available literature is limited to case reports about HIV-positive RA patients with reported side effects. These side effects range from no opportunistic infections (OIs) in some to acute respiratory distress syndrome (ARDS) and disseminated intravascular coagulopathy (DIC) reported in others. Some HIV cases may initially present with rheumatological manifestations. With growing epidemiologic evidence of frequent joint manifestations in HIV-positive patients, HIV testing should be done more frequently in patients with RA, even those who deny risk factors for HIV. This review may help develop future guidelines on how to manage HIV-positive RA patients.

## Introduction and background

Center for Disease Control and Prevention (CDC) reported around 54.4 million adults who were diagnosed with rheumatological diseases between 2013 and 2015 in the US [1]. An estimated total of $139.8 billion (range $135.9-$157.5 billion) national arthritis-attributable medical expenditure reached up to 11% (range 11-13%) of overall medical expenditures [1].

Rheumatological diseases include various diagnoses such as rheumatoid arthritis (RA), fibromyalgia, seronegative spondyloarthropathies such as psoriatic arthritis (PsA), ankylosing spondylitis, and reactive arthritis. They adversely affect the functional capabilities of the patients and are a substantial socioeconomic burden. Management of these patients seems challenging with co-morbidities and chronic infections with hepatitis B virus (HBV), hepatitis C virus (HCV), and human immunodeficiency virus (HIV). HIV has the capability of causing an acute stage of infection and remain in a chronic persistent stage of replication [2]. Recent research showed arthritis in more than 5% of HIV patients and may reach up to 12% [3]. HIV-infected patients have shown a higher risk of rheumatologic disease incidence in some cases [4]. A study mentioned that up to 15% of HIV patients can have a low to medium level of anti-cyclic citrullinated peptide antibodies (anti-CCP antibodies) [3].

Controlling the symptoms of RA in HIV-positive patients remains a challenge because of the lack of available options in patients not responding to the conventional disease-modifying anti-rheumatic drugs (DMARDs). Also, prolonged use of corticosteroids in immunosuppressed HIV patients may cause the HIV infection's progression and cause uncontrolled viral replication. Biological agents such as infliximab, adalimumab, and etanercept have scarce information about its safety and efficacy in HIV patients. The fact that biologics inhibits the pro-inflammatory cytokine tumor necrosis factor-alpha (TNF-α) raises a concern about their use without predisposing the patient to opportunistic infections (OIs) [5,6]. The use of infliximab and etanercept is associated with increased susceptibility to intracellular pathogens such as *Mycobacterium tuberculosis*, atypical mycobacteria, and endemic fungi such as *Histoplasma capsulatum*, *Coccidioides immitis*, yeasts such as *Cryptococcus neoformans* and Candida, *Pneumocystis pneumonia*, and *Listeria monocytogenes* [5,6].

TNF-α is a pro-inflammatory cytokine produced from monocytes in innate and adaptive immunity secreted in response to bacterial lipopolysaccharides when cleaved by a specific metalloproteinase released from the surface of the cell. Two different types were first isolated from animals in 1984, which were also found to regress tumor cells’ growth. As of 2003, FDA (US Food and Drug Administration) has approved three TNF-α inhibitors (infliximab, adalimumab, and etanercept) [6]. They were later found to have a pivotal role either by apoptosis or cellular growth and differentiation [7]. In some HIV-1 infection cases, the serum concentration of TNF-α released from mononuclear phagocytes and alveolar macrophages was elevated [8].

Very little information is available about treatment with biologic agents in HIV-positive patients with rheumatological disease. In this review, we tried to gather more information and answer questions regarding its safety, efficacy, side effects, and complications in HIV patients using this treatment option.

## Review

After the invention of the highly active antiretroviral treatment (HAART), HIV became a chronic multisystemic disease. Both rheumatologic diseases and HIV require a prolonged course controlled with a variety of medications. Unfortunately, with overlapping manifestations like joint manifestations as an initial presentation of HIV, it can be a dilemma with co-existing HIV infection. Using biological agents such as anti-TNF agents that act on cell-mediated immunity in patients with RA after DMARD failure can be risky. Factors like patient’s occupational exposure, medical history, a cluster of differentiation-4 +T- cells (CD4 + T-cells) count, viral load levels, and endemic infections are of significant importance and should be considered while managing these patients [9]. The scarcity of information regarding the use of these agents in HIV-patients is the reason for hesitancy for clinicians to proceed to this next step after unresponsiveness to DMARDs in rheumatological patients [10].

Mechanism of action

The TNF was first discovered as a glycoprotein released in response to the Cachectin endotoxin with killing cancerous cells as a benefit. It is produced by monocytes, macrophages, natural killers, and T helper cells as a cytokine for T helper 1 (Th1) cells [11,12]. It is part of the proinflammatory response and produces interleukin-1β (IL-1β) and interleukin-6 (IL-6), together with its receptors, tumor necrosis factor receptor 1 (TNFR1) and tumor necrosis factor receptor 2 (TNFR2), which are involved in chronic inflammatory diseases and carcinogenesis [11,12]. The TNF exerts its cellular effect through TNF receptors. The receptors consist of an extracellular domain common to all subtypes, with TNFR1 possessing a death domain and TNFR2 not having this domain. In a trial to understand the role of TNF interaction with its receptor in the HIV-1 entry, in-vitro studies about macrophages treated with HIV-1 gp120 (glycoprotein 120) envelope protein found a delay in HIV DNA long-terminal repeat detection (LTR) [11,12]. That was also pretreated with TNF concomitantly, best explained by CD4 receptor and C-C chemokine receptor type-5 (CCR5) downregulations on the cell surface. The downregulation resulted from granulocyte-macrophage colony-stimulating factor (GM-CSF) release following macrophage stimulation with TNF, inhibiting the entry of strains that are CCR5 dependent. Also, the interaction between TNF and TNFR2 was found to release HIV suppressive mediators such as nuclear factor kappa-light-chain-enhancer of activated B cells (NF-KB), macrophage inflammatory protein-1 beta (MIP-1β), and macrophage inflammatory protein-1 alpha (MIP-1α). These factors lead to further CCR5 downregulation, indicating that the TNF role in inhibiting the HIV entry is mediated through TNFR2, not TNFR1, as CD4 downregulation in TNF-treated macrophages occurs at the transcription level [11,12].

As TNF binds to TNFRs, it activates NF-KB (promoting cell survival), c-Jun N Terminal Kinase (JNKs), and caspases (promoting cellular death), p65/p50NF-KB, and AP-1 (activator protein-1) complexes to the transcription factors present in HIV-1 LTR promoter. Thus, it mediates NF-KB translocation into the nucleus, with subsequent LTR stimulation activating HIV-1 in chronically infected T-cells. TNF-α stimulation subsequently triggers some intracellular pathways, including apoptotic ones. Binding of TNF to its receptor activates TNFR- associated death domain (TRADD), which later on binds to Fas-associated death domain protein (FADD) and intern trigger cellular apoptosis via caspases [12].

The virus proteins exhibit some molecular mimicry to TNF signaling, especially in HIV-infected macrophages. The first protein is viral protein r (Vpr), a virion-associated protein that helps the viral integration to the nucleus. This protein also helps establish HIV in infected cells through an anti-apoptotic effect while inducing the apoptotic effect in the surrounding tissues. It also exerts an inhibitory effect on the mitochondria. Its importance lies in HIV replication in T cells and macrophages. The Vpr stimulates NF-KB in T-cells, macrophages, and promonocytic cell line U937. Also, through toll-like receptor 4 (TLR4) and IL-6 secretion, it stimulates HIV production; their role is heavily dependent on the cell type involved.

Another protein that helps in the viral establishment is HIV-negative regulatory factor (Nef). Its recombinant form was found to stimulate cytokines' expression and release mediated by NF-KB activation in culture monocyte-derived macrophages. When rNef was added to chronically infected U1 cell lines, it increased HIV-1 replication. Another important effect of rNef is its ability to induce phosphorylation of key-signaling molecules [13].

Management challenges

Infections Encountered With Immunobiological Agents

The critical TNF-α role in connecting the immune cells monocytes, neutrophils, B cell, and T cell function when blocked, has been associated with various infections. Treatment with TNF-α blockers has been associated with numerous infectious agents such as *M. tuberculosis* or *Avium intracellulare*, other bacteria such as *L. monocytogenes*, and Streptococcus pneumoniae, as well as fungi such as *Candida albicans*, *Pneumocystis jiroveci*, *Aspergillus fumigatus*, *Histoplasma capsulatum*, *Cryptococcus neoformans*, and *Coccidioides immitis* [8].

One of the earliest papers was the case report by Kaur et al., which discussed a case of an RA patient with HIV, HBV, and HCV who received etanercept with being concurrently treated with HAART therapy. The patient displayed an increase in the absolute CD4 count up to 530/mm^3^ while his viral copies decreased to less than 50 copies/ml. The patient symptoms later were controlled on etanercept with a decreased number of involved joints from 28 to 20 joints and to less than five after three months; the patient did not face any adverse events or OIs [14].

Also, another prospective study by Walker et al. studied the effect of a chimeric monoclonal antibody on inhibition of TNF-α. The study involved six HIV-1 patients, selected with CD4+ cell <200/mm^3^ after two months of nucleoside analogs treatment. After two infusions which were 14 days apart, serum TNF-α fell with no marked changes in plasma HIV ribonucleoprotein (RNA) or CD4 cell counts [8].

A case report by Liang et al. discussed the usage of etanercept in an RA patient with HIV-positive status. The patient had symmetrical polyarticular involvement and showed no improvement with DMARDs. The patient improved markedly after etanercept use and kept using it for two years with no OIs reported [5].

On the other hand, a case report by De Nardo et al. reported septic shock in RA patients on etanercept after receiving the Influenza vaccine. The patient was vaccinated two months after starting etanercept as mandated by her clinicians due to her medical history of chronic obstructive pulmonary diseases (COPD) and HIV [15]. Her CD4 count decreased to 549 CD4 cells/mm^3^ and undetectable HIV RNA. The patient deteriorated, and developed acute respiratory distress syndrome (ARDS) and disseminated intravascular coagulopathy (DIC). The patient improved after intubation and mechanical ventilation in the intensive care unit. Marked hypogammaglobulinemia was found and intravenous immunoglobulin was given until the patient improved [15].

In a case series report by Chin-Hong, the use of infliximab in an HIV-positive patient was associated with the occurrence of OI of *P. jiroveci* infection [9]. The infection occurred with a higher CD4+ count (above 240) than the suspicious level of Pneumocystis infection in HIV patients, which was thought to be due to TNF-α use with HIV infection.

Another retrospective cohort study, done by four centers in the USA (Cleveland Clinic, Johns Hopkins Hospital, University of Miami, and Brigham and Women’s Hospital) aimed to identify the incidence of serious infections in HIV-patients treated with TNF-α inhibitors after being diagnosed with HIV. The incidence of serious infections in patients with viral load >500 copies/mL was slightly higher than those with a viral load of ≤500 copies/mL, but not significantly different [16]. However, it was within the range of data obtained from the Curtis et al., which was a strong prospective cohort study involving thousands of RA patients comparing the outcome in between different groups with different demographics, comorbidities, and treatment modalities in different US and European RA registries [16]. Table 1 shows the infectious complications interpreted in some studies.

**Table 1:** Infectious complications encountered with the biological agents used by different papers TNF-α: tumor necrosis factor-alpha, OI: opportunistic infection, HCV: hepatitis C virus, HIV: human immunodeficiency virus, CD4: cluster of differentiation-4.

Author	Year of publication	Purpose of the study	Type of study	Result/conclusion
Chin-Hong [9]	2019	Study the infectious and other complications in HIV-patients treated with immunobiological agents.	Case-series	Use of infliximab in an HIV-positive patient was associated with the occurrence of OI of P. jiroveci infection even with higher CD4+ count (above 240) than the suspicious level of Pneumocystis infection occurrence in HIV patients, which was thought due to TNF-α use with HIV infection.
Wangsiricharoen et al. [10]	2017	Study the incidence of serious infections in HIV-infected patients who received TNF-α inhibitor therapy for concomitant autoimmune diseases among different stratified viral loads.	Retrospective cohort	The serious infections’ rate in patients with viral load >500 copies/ml at the beginning of therapy was slightly higher than those with viral load ≤500 copies/ml, but not significantly different.
Walker-Bone et al. [11]	2016	Management of musculoskeletal disorders among patients living with HIV.	Review article	Few data published, with different outcomes ranging between improvement of one patient with HIV and HCV co-infection in one patient and discontinuation due to OIs in other case reports.

Non-Infectious Adverse Consequences of TNF-α Blockers

Lymphoma and solid tumors: Lymphoma was not reported in RA patients treated with biological agents than RA patients who did not receive it. Despite that several studies that show there is no evidence of increased risk of solid tumors, a recent meta-analysis for Bongartz et al. [17], shows a higher rate of malignancies in HIV-positive patients with RA treated with biological agents.

Congestive heart failure: Although some clinical trials showed worse prognosis with increasing the TNF-α blockers, the overall conclusion showed no need to screen for heart failure with echo unless the patient has a history of heart failure. However, if the patient has a mild congestive heart failure (CHF), New York Heart Association (NYHA) class I or II should have a baseline echo. Close monitoring is required for those patients but biological agents should be avoided in CHF NYHA class III or IV [18,19].

Neurological: The incidence of demyelinating diseases is not higher than the general population even though some post-marketing surveillance showed some cases of demyelinating disorders; however, no causal association can be established due to limited data [18].

Hematological: Incidence of any blood dyscrasias is rarely reported, however, if any patient shows signs of easy bruising, pallor, fever, or gum bleeding should stop the biologic agent immediately [18].

Glomerulonephritis: Very few cases of glomerulonephritis were reported with TNF-α inhibitors; discontinuation of these agents with steroids and immunosuppressive medications leads to improvement [18].

Is it HIV presenting with arthritis or is it a rheumatological disease with comorbid HIV infection?

HIV was found to be presenting with arthritis at any stage of the infection independent of HAART, either by symmetrical polyarticular, oligoarticular, or monoarticular [2,3]. Asymmetric oligoarticular was the most common pattern especially involving the knees and ankles joints. There were less evident mucocutaneous manifestations or enthesopathy with p24 antigen, HIV DNA, and core viral HIV protein found in the affected joints’ synovial fluid [11]. There is epidemiological evidence of the growing prevalence of rheumatic diseases in HIV positive patients. Also mentioned in the paper by Fox and Walker-Bone [19], that HIV-infected cases in the post-HAART era develop symmetric polyarthritis of the small joints of the hands and feet, clinically similar to RA. The immune system change happening with HIV was found to improve the clinical presentation of already diagnosed RA patients. On the other hand, some HIV-positive patients developed de novo RA disease as a part of immune reconstitution syndrome after HAART initiation, which was approved to be of lower prevalence in other retrospective cohort study by Yang et al., in a Taiwanese cohort followed for 20 years [20]. Still, generally, RA develops when HIV is controlled with undetectable viral activity and a CD4+ count >200 cells/mm^3^ [11,14,15].

For reactive arthritis, HIV positive patients have demonstrated a more prominent skin involvement than HIV-negative patients, as psoriasiform rashes, guttate, inverse, and erythrodermic subtypes of psoriasis were more florid compared to seronegative patients reported by Caroll et al. [3]. In contrast, axial involvement and uveitis were less common than HIV-negative patients. PsA was reported first as a symptom of end-stage acquired immune deficiency syndrome (AIDS), with a prevalence estimated as 0.25% in the US, which explains a finding that HIV increases the risk of acquiring PsA. However, those findings were contradicting the other two large case-control studies in the early 1990s by Hochberg et al. and Clark et al. [21,22], also, another longitudinal study by Calabrese et al. [23], over 11 years of follow-up that found only three incident cases among 395 HIV positive cases with an estimated incidence rate approximate to that of the estimated population of 0.05%.

Another acute HIV infection phenomenon is a syndrome called painful articular syndrome in which patients had severe arthritis without subjective synovitis and short duration (224 hours). It is thought to be associated with HIV infection acquired through intravenous drug use [14,15,20]. It usually presents in one large joint with severe pain requiring emergency service and opiates use. Berman et al. described it in 1988 with a 10% prevalence among US patients from the US and Argentina [24]. In an Indian paper by Kumar Kole et al., the syndrome was mentioned together with low back pain as a major cause of distress in HIV patients and absence from work, myalgia was also reported as the most common symptom presenting in 46% of patients [25].

Diffuse infiltrative lymphocytosis syndrome (DILS) was described as a disease entity similar to systemic sclerosis affecting salivary glands causing swelling and sicca symptoms, associated with hypergammaglobulinemia, commonly, but not necessarily presenting with bilateral parotid glands enlargement, as extra-articular manifestations also occur as pneumonitis, myositis, neuropathies, nephropathy, hepatitis, and uveitis. Diagnosis heavily relies on histopathological examination confirming lymphocytic infiltration in the absence of anti-Ro/SSA and anti-La/SSB and more cluster of differentiation 8+ T cells (CD8+ T) infiltration than CD4+, with symptoms improving after the introduction of HAART into the treatment plan [11].

In another case-control study by Berman et al. [26], reported myalgia in HIV-infected cases is twice as common as in the HIV-negative control group.

As a result, in some dermatological diseases such as recalcitrant psoriasis or rheumatological manifestations such as sexually transmitted reactive arthritis, keratoconjunctivitis sicca syndrome in the absence of anti-Ro, anti-La antibodies, vasculitis should ignite a lower threshold for HIV testing [11].

Biological agents have started to be used in some individual cases. Do we need to develop some guidelines to use it in HIV-positive patients? Do we need to study a cutoff point of viral load and HIV antibody levels to start it more extensively in rheumatological patients with HIV? Do we need to have a low threshold to test for HIV in patients presenting with arthritis? Table 2 shows the results of biological agents used in numerous studies.

**Table 2:** The results of biological agents use in different papers HAART: highly active antiretroviral treatment, RA: rheumatoid arthritis, TNF-α: tumor necrosis factor-alpha, CD4+ T: cluster of differentiation-4 +T, RNA: ribonucleoprotein.

Author	Year of publication	Purpose of the study	Type of study	Result/conclusion
Liang et al. [5]	2017	Use of etanercept to treat RA in an HIV-positive patient	Case report	Excellent response to etanercept for 30 months without side effects with Initial CD4+ T cells count of 418, and viral copies/ml of 20 which turned into 516 and less than 20 at the end of the treatment period.
Carroll et al. [3]	2016	Management challenges in RA patients with HIV	Review	Biological agents used in HIV patients was safe as long as HIV is well controlled by HAART.
Adizie et al. [4]	2016	Inflammatory arthritis in HIV patients	Review	Biologic agents were successful in the treatment of HIV positive rheumatic patients and no adverse events.
Yang et al. [20]	2015	Rheumatological disease prevalence in HIV patients on HAART	Retrospective study	The prevalence of rheumatic diseases in the HIV-1 cohort was similar to the general Taiwanese population.
De Nardo et al. [15]	2013	Septic shock occurrence after seasonal influenza vaccination in HIV-infected patient during Etanercept use in RA patients	Case report	Multiple organ failure occurrences after Influenza vaccination in HIV positive patient with RA.
Kaur et al. [14]	2007	Successful etanercept use in an HIV-positive patient with RA	Case report	Improvement in RA patient symptoms with HIV infection and a decrease in the number of tender swollen joints from 28 to 20 after three months of therapy.
Calabrese et al. [2]	2004	Safety of anti-TNF in patients with chronic viral infections	Review article	Data are not sufficient, only a few cases are reported but successfully treated.
Walker et al. [8]	1996	Effect of TNF-α Monoclonal antibody injection two weeks apart and its effect on TNF, CD4+ T cells, and HIV viral RNA levels	Prospective study	TNF-α successfully inhibited TNF-α in HIV-1 patients.

## Conclusions

We lack clear guidelines to deal with challenging rheumatological conditions in HIV-positive patients. The reviewed articles showed that both conditions may frequently co-exist. When treated with DMARDs and biological agents, some patients respond better and tolerate these medicines well as compared to others. A few patients had severe adverse effects after the use of DMARDs, including coma. Having a cut-off point of viral load and CD4+T-cell count can guide physicians about starting biological agents if they do not respond to DMARDs. More clinical trials are needed to reach a consensus regarding when the biological agents should be started.
